# Meeting report: Signal transduction meets systems biology

**DOI:** 10.1186/1478-811X-10-11

**Published:** 2012-04-30

**Authors:** Christine Louis-Dit-Sully, Katharina F Kubatzky, Jonathan A Lindquist, Christine Blattner, Ottmar Janssen, Wolfgang W A Schamel

**Affiliations:** 1 Max Planck Institute of Immunobiology and Epigenetics and Biology III, Faculty of Biology, University of Freiburg, 79108, Freiburg, Germany; 2 Department of Infectious Diseases, Medical Microbiology and Hygiene, University Hospital of Heidelberg, Im Neuenheimer Feld 324, 69120, Heidelberg, Germany; 3 Institute of Molecular and Clinical Immunology, Otto-von-Guericke University, Leipziger Strasse 44, 39120, Magdeburg, Germany; 4 Karlsruhe Institute of Toxicology and Genetics, PO-Box 3640, 76021, Karlsruhe, Germany; 5 Christian-Albrechts-University of Kiel, Institute for Immunology, UKSH Campus Kiel, Arnold-Heller-Str. 3 Bldg 17, 24105, Kiel, Germany; 6 Center for Biological Signalling Studies BIOSS, University of Freiburg, 79104 Freiburg and Center for Chronic Immunodeficiency CCI, University Clinics, 79106, Freiburg, Germany

## Abstract

In the 21^st^ century, systems-wide analyses of biological processes are getting more and more realistic. Especially for the in depth analysis of signal transduction pathways and networks, various approaches of systems biology are now successfully used. The EU FP7 large integrated project SYBILLA (Systems Biology of T-cell Activation in Health and Disease) coordinates such an endeavor. By using a combination of experimental data sets and computational modelling, the consortium strives for gaining a detailed and mechanistic understanding of signal transduction processes that govern T-cell activation. In order to foster the interaction between systems biologists and experimentally working groups, SYBILLA co-organized the 15th meeting “Signal Transduction: Receptors, Mediators and Genes” together with the Signal Transduction Society (STS). Thus, the annual STS conference, held from November 7 to 9, 2011 in Weimar, Germany, provided an interdisciplinary forum for research on signal transduction with a major focus on systems biology addressing signalling events in T-cells. Here we report on a selection of ongoing projects of SYBILLA and how they were discussed at this interdisciplinary conference.

## Introduction

As cellular constituents of the adaptive immune system, T cells carry an individual T cell antigen receptor (TCR)/CD3 complex with which they recognize specific antigens, resulting in activation of the cell and the mounting of an immune response [1,2]. However, to initiate a successful immune response against pathogens, without creating an inappropriate response against self-antigens, T cells have to discriminate between healthy cells of the body and diseased or infected cells. It is thought that the affinity of antigens to the TCR/CD3 complex governs this discrimination during intrathymic development. In the periphery, infected and diseased cells will present specific ‘foreign’ antigens with high affinity to the TCR leading to activation of the T cell. Due to the selection process, peripheral self-antigens have low or no binding-affinity and should not result in T-cell activation, but might rather be involved in T-cell survival. If the multiple backup systems of central and peripheral tolerance fail, T cells with high affinity to self-antigens might cause autoimmunity.

T-cell activation is a complex process relying on multiple layers of tightly controlled intracellular signalling modules that form an intricate network. In order to gain systems-level insight into critical modules of the network and finally into the behaviour of the complete network, the SYBILLA consortium was founded. It groups 18 partners from 9 different EU countries, including a management company (Novamen, Lyon, France; represented by Sandrine Rival in SYBILLA), and coordinated by Wolfgang Schamel (Freiburg, Germany). Detailed information can be found at http://www.sybilla-t-cell.de.

The current development of several ongoing projects has been reported at the meeting and will be described below. In essence, through a multidisciplinary effort, SYBILLA aims to understand at the systems level, how T-cells discriminate foreign from auto-antigens, how T cells differentiate from naive cells into effector cells and how T-cells react to auto-antigens in case of autoimmune diseases.

To meet its goals SYBILLA started with improving and standardizing new quantitative and high-throughput technologies to obtain quantitative data on signalling networks. These include multicolour immunoprecipitation measured by flow cytometry (IP-FCM) [3], with which accurate multidimensional data on protein-protein interactions and phosphorylations can be acquired. Improved mass spectrometry-based strategies were developed by Rudi Aebersold and Matthias Gstaiger (both SYBILLA in Zürich, Switzerland) for a systematic and quantitative analysis of protein interactome and phospho-proteome changes upon cell stimulation [4]. Finally, novel phospho-specific antibodies are generated by the antibody-producing company EXBIO praha (SYBILLA near Prague, Czech Republic) [5].

**Figure 1:**
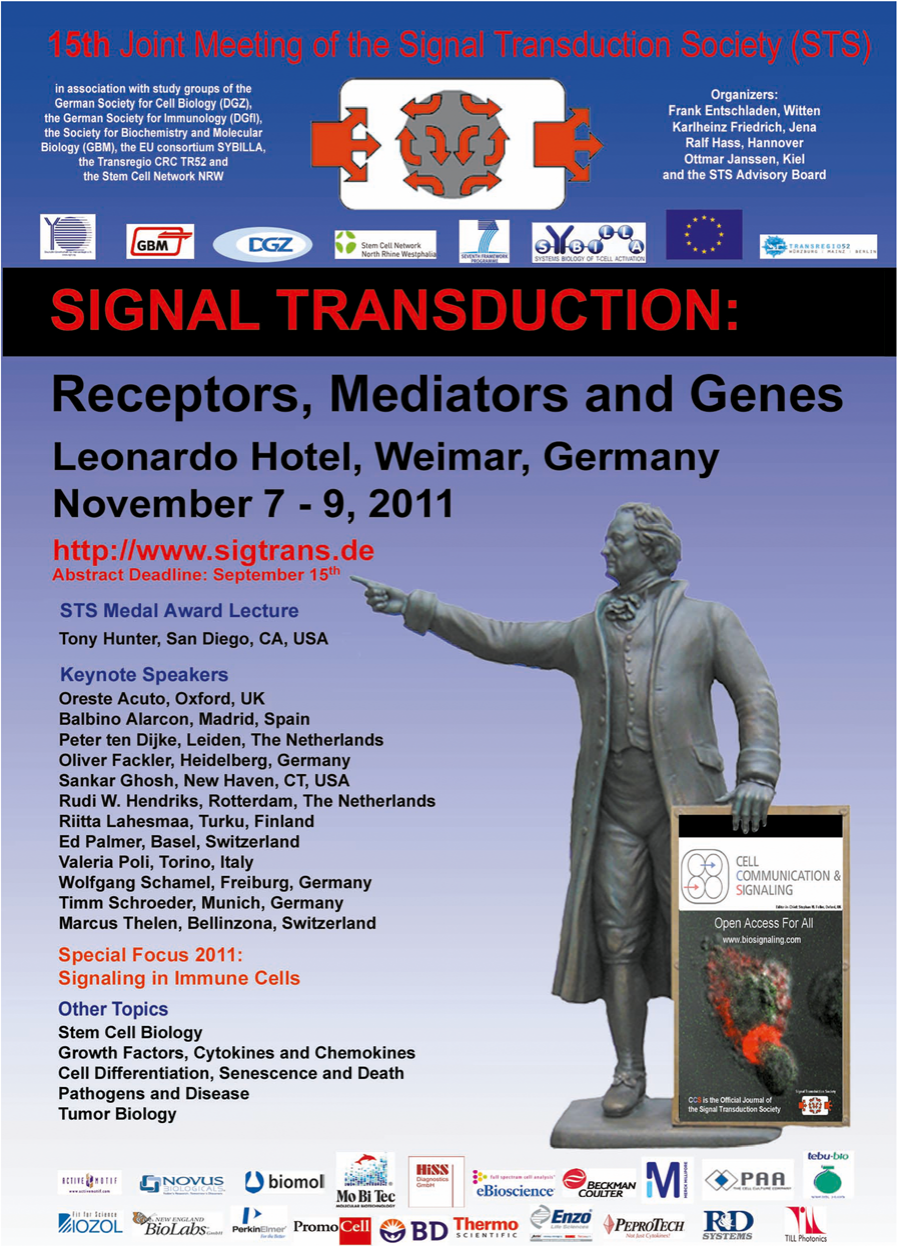
**Poster of the 15**^**th**^** STS meeting.** The 15^th^ meeting of the Signal Transduction Society (STS) “Signal Transduction: Receptors, Mediators and Genes” in Weimar, Germany was co-organized by the EU FP7 consortium SYBILLA.

Early on, SYBILLA sought to have a competent partner to exchange ideas and concepts about signal transduction processes. With the Signal Transduction Society (STS), SYBILLA found the ideal partner and has for example published the stoichiometry of the TRIM-containing TCR/CD3 in the STS journal *Cell Communication and Signaling*[6,7]. In 2011 SYBILLA, the STS and the Study Group Signal Transduction (AKS) of the German Society for Immunology (DGfI) (chair: Ottmar Janssen, Kiel) decided to co-organize a focus symposium on “Signaling in immune cells” at the annual STS conference “Signal Transduction – Receptors, Mediators and Genes” [8,9] (Figure 1). Thus, the 2011 STS conference held in November in Weimar, Germany, set the stage for a so far unique get together of scientists working on different aspects of signal transduction that converged at the focus on T-cell activation. Here we provide a brief meeting report and – at the same time – present several of SYBILLA’s recent achievements. 

## Early events in T-cell signalling

**Figure 2:**
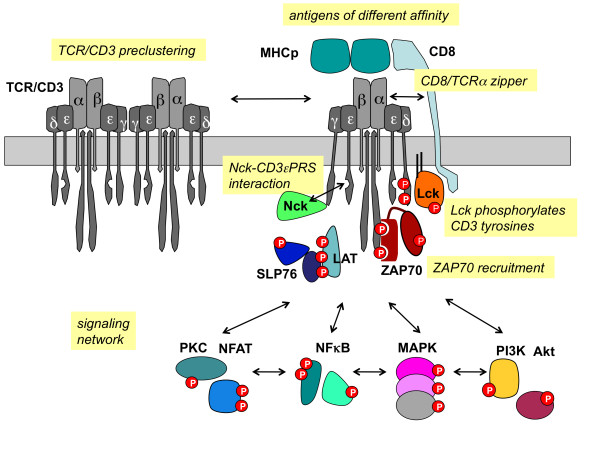
**TCR/CD3 signaling.** The intracellular network of T-cell signalling with a focus on the early, TCR/CD3-proximal events is shown. The processes mentioned in the text are marked with text in italics. Red circles with a P are phosphorylation sites.

The first element of the intracellular T-cell signalling network is the TCR/CD3 complex itself that not only recognizes the antigens but can also distinguish between low and high affinity antigens [10]. Thus, one focus of SYBILLA is to characterise and mechanistically understand the differential signal initiation by the TCR/CD3 complex. During the STS meeting in Weimar, Balbino Alarcon (SYBILLA in Madrid, Spain) presented new data showing that the proline-rich region in the cytoplasmic tail of the CD3ϵ subunit is crucial for T cell activation (Figure 2). Upon antigen-binding, this region is exposed and binds to the adaptor protein Nck [11,12]. His group generated mice with a mutation in the CD3ϵ tail, thereby preventing Nck association, and also mice with another mutation in CD3ϵ which disables the exposure of the proline-rich region. In both cases, T cell functions *in vivo* were defective [13]. 

Besides structural changes in CD3ϵ, a more precise knowledge on the overall structure of TCR/CD3 complex is crucial. Balbino Alarcon (Madrid, Spain), Wolfgang Schamel and Burkhart Schraven (SYBILLA in Magdeburg, Germany) have collaborated to decipher the stoichiometry of the TCR/CD3 [7,14] and to obtain a first 3D model of the complete TCR/CD3 complex [15]. At the meeting, Wolfgang Schamel presented collaborative data with Balbino Alarcon showing that pre-clustering of the TCR/CD3 complex, which takes place independently of antigen-binding [16,17], enhances the sensitivity of T-cells [18]. Interestingly, naïve T-cells possess mostly monovalent TCR/CD3 complexes whereas effector and memory T cells express pre-clustered TCR units and therefore display lower signal thresholds and are easier to activate (Figure 2). Moreover, Schamel showed that the lipid composition of the plasma membrane is critical for TCR/CD3 pre-clustering. Lipids segregate into raft and non-raft microdomains [19-21] and raft-associated lipids are the ones that enable/cause this pre-clustering. In line with this, Tapio Lönnberg from Riitta Lahesmaa’s group (SYBILLA in Turku, Finland) presented his lipidomics data showing that T cells change their membrane lipid composition upon differentiation from naïve to effector T cells.

**Figure 3:**
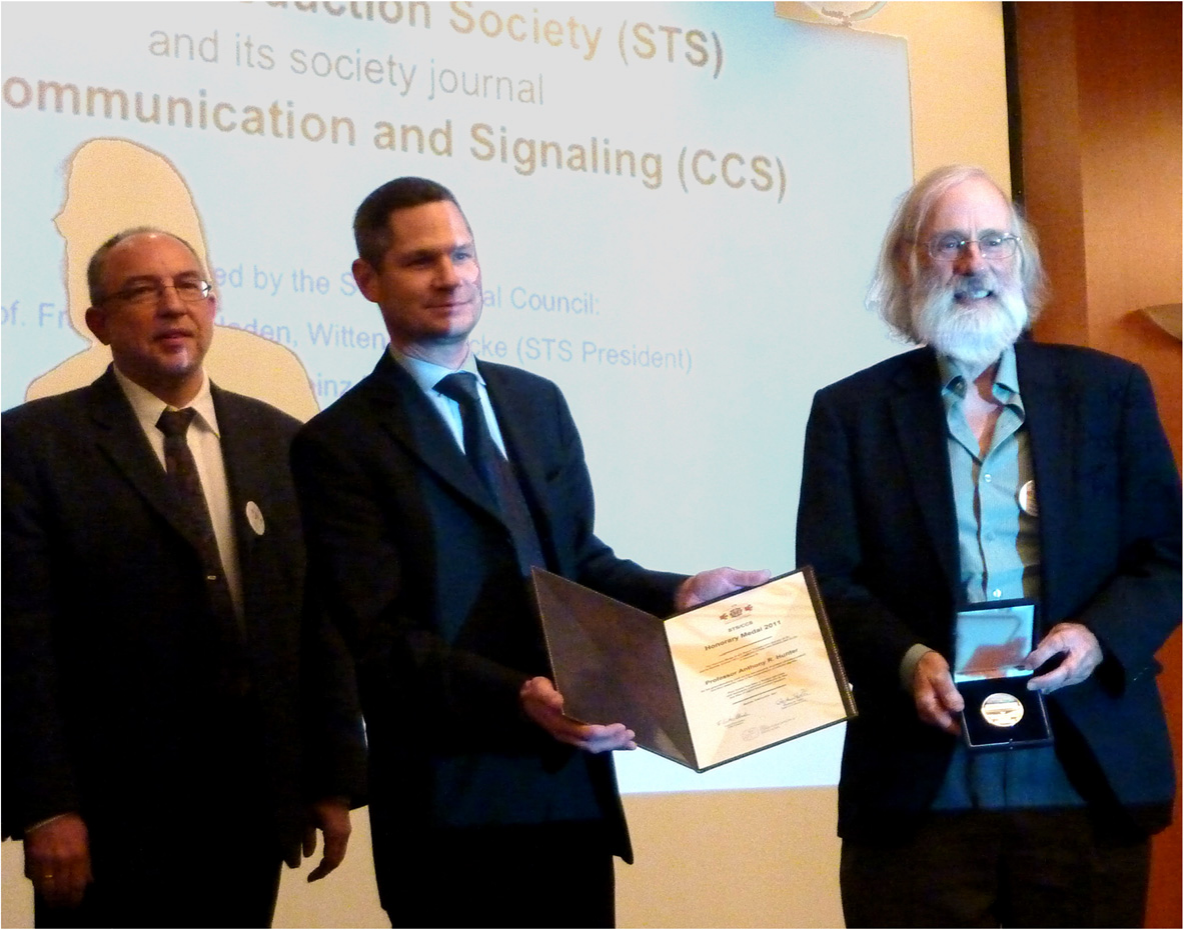
**Tony Hunter receives the STS/CCS Honorary Medal.** Since 2010 the Signal Transduction Society (STS) and *Cell Communication and Signaling* honour outstanding researchers in the field of signal transduction research with this medal. 2011 Tony Hunter received this award for the discovery of tyrosine phosphorylation and other important contributions in cell signaling research.

Antigen-recognition by the TCR/CD3 complex leads to the phosphorylation of tyrosines in the cytoplasmic tails of CD3. The scientist who first discovered tyrosine phosphorylation as a fundamental mechanism for signal transduction was a special guest in this year’s conference. Since 2010, the STS and *Cell Communication and Signaling* honour researchers for outstanding contributions to signal transduction research with the “STS/CCS Honorary Medal”. The first medal was awarded to Anthony J. Pawson (Toronto, Canada) in 2010 [22]. In 2011, Tony R. Hunter (La Jolla, CA, USA) was honoured for the discovery of protein tyrosine phosphorylation (Figure 3). He described during his talk how the tyrosine kinase field exploded after his initial, almost accidental, detection of phosphorylated tyrosine in Polyoma antigen immunoprecipitations in 1977 [23]. 

Concerning the phosphorylation of the TCR/CD3 subunits, an exciting and stimulating controversy on the activation of the tyrosine kinases Lck and Fyn, that phosphorylate CD3, is taking place within SYBILLA and became evident also at the STS conference. Burkhart Schraven showed that a subset of the kinase Lck that is associated with the TCR is activated upon TCR stimulation. This could be an important finding contributing to the understanding of how antigen binding to the TCR causes TCR phosphorylation (Figure 2). By contrast, Oreste Acuto (SYBILLA in Oxford, UK) presented data that were generated in collaboration with Antonella Viola (SYBILLA in Milan/Padua, Italy), Thomas Höfer (SYBILLA in Heidelberg, Germany) and Lars Fugger (SYBILLA in Oxford) showing the presence of a dually phosphorylated (pY394 and pY505) and active pool of Lck even in resting T-cells [24]. In contrast to Burkhart Schraven, he proposed that Lck is not activated upon TCR/CD3 stimulation. Jonathan Lindquist (SYBILLA in Magdeburg, Germany) presented the surprising finding that PAG/Cbp-depleted human T cells which enhance Fyn activity (next to Lck the second Src-family kinase that can phosphorylate CD3) become anergic. This could be explained by Fyn-dependent hyper-phosphorylation of the inhibitory receptor CTLA-4. Clearly, further research is needed to clarify how antigen-binding to the TCR/CD3 causes CD3 phosphorylation.

## Mathematical models of early T-cell signalling

Deciphering the mechanisms of how the TCR/CD3 complex is activated, is a starting point to understand how this receptor can distinguish between low and high affinity antigens. This needs close collaboration between experimentalists who generate quantitative data sets and applied mathematicians who use deterministic and stochastic approaches to describe those data [25-27]. Fostering those interactions is one goal of SYBILLA.

Based on the already discussed finding that antigen-binding to the TCR/CD3 complex exposes the proline-rich region in CD3ϵ and that this exposure is necessary for T-cell activation [28,29], Thomas Höfer and Wolfgang Schamel have formulated a mathematical model that describes these events. The model was parametrized and validated using extensive biochemical data, such as IP-FCM (see above). Together with functional data obtained by Balbino Alarcon and Ed Palmer (SYBILLA in Basel, Switzerland), the model shows that exposure of the proline-rich region has the property to distinguish between antigens of different affinities.

T-cell antigens bind simultaneously to the TCR/CD3 complex and the co-receptor CD8. Extending this observation, Ed Palmer presented his TCR-CD8 zipper model, in which low affinity ligands bind shortly to the TCR/CD3 and CD8, thereby not allowing CD8 to zip (bind) to the TCR/CD3 (Figure 2). High affinity ligands, however, bind longer to the TCR/CD3 complex, enabling CD8 to form a stable complex with the TCR in which the CD8-bound kinase Lck can phosphorylate TCR/CD3 subunits and initiate signaling [30]. This model belongs to the kinetic proofreading models [31], in which the duration of the antigen-TCR/CD3 interaction determines the outcome.

Finally, Anna Schulze (from Thomas Höfer’s group) presented a new mathematical model of TCR/CD3 phosphorylation and ZAP70 recruitment (Figure 2) that was based on IP-FCM data generated by Wolfgang Schamel’s group [3]. These accurate time-resolved data allowed for the first time the quantitative determination of phosphorylation and dephosphorylation rate constants in the mathematical model, indicating very rapid turnover of both ITAM and ZAP-70 phosphorylations. In contrast to the previous proposal that multiple phosphorylations at the TCR complex kinetically proofread ligand quality (see e.g. [25][31]), the actual parameter values indicate that such a mechanism can only play a minor role in ligand discrimination.

## Holistic views on T-cell activation

Besides the TCR/CD3 and associated kinases, the LAT/SLP76 signalosome (Figure 2) is examined in detail within SYBILLA by Oreste Acuto and Bernard Malissen (SYBILLA in Marseille, France). This topic was not covered at the conference and thus, we would like to refer to the literature [32,33]. The PKC/NFAT module is studied by Gottfried Baier (SYBILLA in Innsbruck, Austria). Natascha Hermann-Kleiter of his group reported on the NR2F6 receptor that specifically dampens NFAT-mediated IL-17a promoter activation in CD4 T lymphocytes. Dysregulation of this mechanism seems to play a role in the development of certain autoimmune diseases [34]. Another important pathway for T-cell activation is the NF-κB pathway that was covered in the conference by several talks (Figure 2). Sankar Ghosh (New York, NY, USA) was invited by the Transregional Collaborative Research Center TR52 "Transcriptional Programming of Individual T-Cell Subsets" (chair: Edgar Serfling, Würzburg, Germany) and gave an introductory keynote lecture in which he discussed the role of NF-κB as a molecular switch of lymphocyte development and functions. In particular, he suggested that PDK1 is a scaffold for PKCθ and Carma1, contributing to NF-κB activation. Rebecca Breuer (Heidelberg, Germany) reported about the phosphatase PP2R5C that controls NF-κB activation and Sarah Jill de Jong (Erlangen, Germany) showed that the viral oncogene Tio also controls T-cell NF-κB signaling.

Next to detailed research on early signalling, comprehensive understanding of the complete T-cell signalling network is another objective of SYBILLA. This is mostly done by transcriptomic and phospho-proteomic analyses with subsequent description by dynamic models.

The importance of using high-through-put methods to understand molecular mechanisms was highlighted at the conference by SYBILLA partner, Riitta Lahesmaa (SYBILLA in Turku, Finland) with a talk on ‘High-through-put biology to understand molecular mechansims of human T helper cell differentiation’. She reported the transcriptional regulation of lymphocyte activation and differentiation. Applying a holistic approach, she profiled gene expression during Th2 cell differentiation. Bioinformatic description of the data by Harri Lähdesmäki (SYBILLA in Turku, Finland) have led to novel hypotheses on the key factors involved in human Th2 cell differentiation. After IL-4 stimulation, STAT6 is needed for the regulation of gene expression controlling human Th2 cell differentiation [35]. In another talk, Yuri Shebzukhov (Berlin, Germany) showed data indicating less active Jnk (a MAP kinase) and concomitantly less c-Jun phosphorylation in Th0 and Th2 cells compared to Th1 and Th17.

The transcriptomics data, provided by Riitta Lahesmaa, will be integrated with phospho-proteome and interactome data, generated within SYBILLA by Bernard Malissen (who has generated knock-in mice for this purpose), Rudi Aebersold and Matthias Gstaiger as well as Oreste Acuto. Signalling network behaviour is studied in primary murine and human T cells in healthy states and is then extended to autoimmune disease models such as multiple sclerosis with Lars Fugger (SYBILLA in Oxford), Pablo Villoslada (SYBILLA in Barcelona, Spain) and diabetes with Arno Hanninen (SYBILLA in Turku, Finland). Finally, spacial data by Antonella Viola’s group complement these approaches [36].

**Figure 4:**
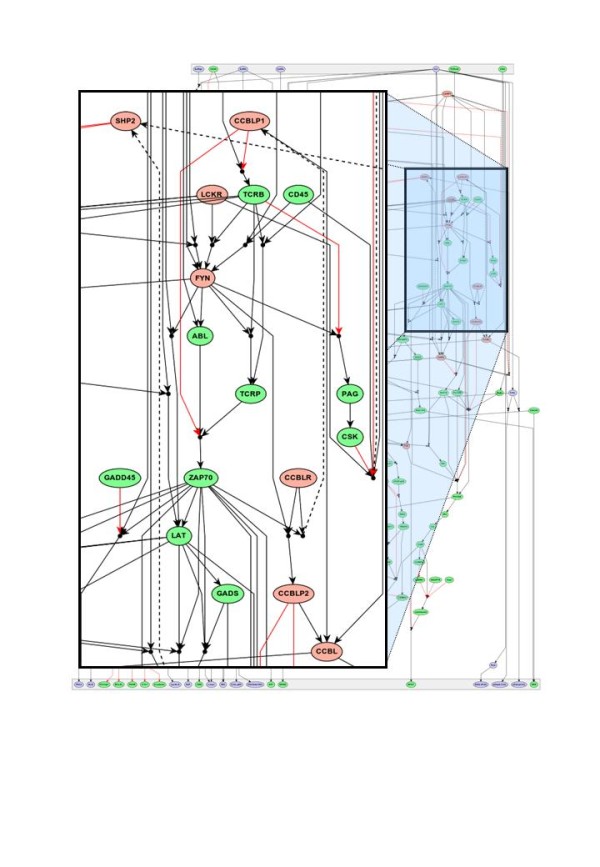
**The merged TCR and IL-2R signaling network.** The top layer represents input nodes and the bottom layer outputs, i.e. molecules including transcription factors that become activated. Solid black arrows indicate activating interactions with black circles denoting AND-connections. For clarity, activating influences with arrows pointing from the bottom to the top are drawn with dashed black lines. Red lines mark inhibitory interactions that are expressed as NOT-conditions in the logical network. Nodes specific to the IL-2R and TCR network are shown in blue and green, respectively. Common nodes are depicted in red. The insert shows a magnified region of the network. Further details can be found in [38].

A future goal is the generation of a “virtual T-cell”; an *in silico* simulation of T-cell activation. Such efforts should help to refine the predictability of physiological and patho-physiological outcomes, as well as to identify new biological markers and new drug targets. For this purpose, a Boolean network of the T-cell signalling network generated by Burkhart Schraven and Jonathan Lindquist [37] is regularly updated within SYBILLA. Indeed, the network was recently extended to integrate signalling through the IL-2 receptor (Figure 4) [38]. Looking for specific therapeutic targets for autoimmune diseases, Friederike Berberich-Siebelt (Würzburg, Germany) suggested some specific roles of SUMOylation of NFAT proteins in multiple sclerosis, while Lucas Kemper from Hansjörg Hauser’s group (Braunschweig, Germany) presented an IFN reporter mouse model showing fast T cell responses to type I IFN in lymphoid tissue. Knowing reported negative effects of type I IFN on T cells, it was surprising not to find alterations in T cell signalling by investigating the endpoints of important signalling cascades (NFAT, NFκB, AP-1). 

As mentioned earlier, T cells are of course also the effector cells of the adaptive immune system. Ottmar Janssen recently characterized the proteome composition of intact effector vesicles from T and NK cells [39-41]. His team identified two morphologically distinct species of cytotoxic effector granulae in T cells characterized by a differential abundance of effector molecules including FasL and granzymes or perforin [42]. Although these vesicles may belong to a common multivesicular body, it appears that they utilize distinct cytoskeletal-associated transport systems for their activation-dependent mobilization to the immunological synapse.

## Intracellular signalling in non-T cells

The strong focus on T-cell signalling of the STS Meeting 2011 was balanced by presentations on signalling events in other cell types. This nicely complemented the otherwise too narrow focus for such a medium-sized international conference.

B-cell signalling was introduced in a keynote talk by Rudi Hendriks (Rotterdam, Netherlands; presentation sponsored by the AKS of the DGfI). He focused on the role of the adaptor protein SLP65, which is a crucial intracellular signal transducer for B cell signaling. Malfunctions of SLP65 can cause immunodeficiencies as well as B cell tumours. Niklas Engels (Göttingen, Germany) identified a tyrosine-based signalling motif within the cytoplasmic tail of the IgG heavy chain, that upon stimulation becomes phosphorylated by Syk and recruits Grb2 to enhance PLCγ activation and calcium flux. New findings on the role of NFATc1 for B cell receptor (BCR)/CD40-triggered proliferation of naive B cells was reported by Anh Thuy Duong Pham (Würzburg, Germany). Using mice with an inactive NFATc1 gene in bone marrow cells and with subsequent conditional re-expression of NFATc1 in NFATc1^−/−^ B cells, she was able to show that NFATc1 supports proliferation after BCR- or CD40-stimulation and that it suppresses the activation-induced cell death of splenic B cells.

Signaling in myeloid cells was addressed by two talks. Thomas Hochdörfer (Aachen, Germany) showed that in mast cells the Cbl-interacting protein CIN85 interacts with SHIP, thereby regulating extent and kinetics of antigen-triggered FcϵR1 internalization. Christopher Tiedje (Hannover, Germany) used macrophages to reveal that p38 (a MAP Kinase)-mediated phosphorylation of an AU-rich element binding factor, TTP, silences TNFα mRNA specifically when translated at the ER. Veronika Jahndel (Heidelberg, Germany) who is investigating how apoptotic cells induce tolerogenic dendritic cells (DCs), identified annexin A1 as an early apoptotic marker on lymphocytes. She could show that annexin A1 interferes with Toll-like receptor signaling, thereby preventing NF-κB activation and DC maturation.

Using a reconstituted system, Iris Behrmann (Luxemburg City, Luxemburg) demonstrated that downstream of the common γ chain, which for example is part of the IL2-R, Jak 1 has a dominant role over Jak3, even though data on Jak3 deficiency had predicted the opposite [43].

## Mechanisms of signal transduction

The STS Meetings traditionally provide a broad and interdisciplinary view on a variety of cellular and organismic signalling aspects, which intentionally provokes to view cellular signalling from different angles and to establish new connections and networks within the community. The topics presented as Workshops included mechanisms of signal transduction in cancer cells, host pathogen interactions, receptor-related studies as well as various aspects of cell fate decisions in immune and cancer cells.

The workshop “Tumor Biology” covered aspects ranging from signalling studies to screening approaches and pharmacologic studies. In her keynote lecture, Valeria Poli (Turin, Italy) discussed functions of the signal transducer and activator of transcription (STAT) 3 protein in inflammation and cancer and the relevance of its subcellular localization for cancer-related signalling events [44]. Arnd Kieser (Munich, Germany) reported on the development of an ELISA-based screen to identify inhibitors of the interaction between viral and cellular signalling molecules and Felix Hausch (Munich, Germany) showed that the interaction of larger FK506-binding proteins with rapamycin contributes to its pharmacological effects. Using a guided clustering approach Alexandra Schrader (Göttingen, Germany) showed data on gene expression modules in Burkitt lymphoma cells. Stephan Feller (Oxford, UK) presented a method that allows the generation of kinome deregulation profiles of cancer cells by label-free quantitative high-throughput mass spectrometry [45] and phosphoproteome analyses by other more conventional techniques.

The workshop “Pathogens and Disease” discussed the role of various viral and bacterial factors in the regulation of signalling cascades in immune cells. A common theme among viral immune evasion strategies is the manipulation of transcription factors or regulators of the actin cytoskeleton such as Rho GTPases. This was nicely illustrated in Oliver Fackler’s (Heidelberg, Germany) keynote talk on the subversion of TCR signal transduction by HIV-1 [46]. As viruses depend on the activity and the survival of their host cell, viral proteins can induce cancer through excess proliferative signals [47]. An example presented by Kristin Katsch (Erlangen, Germany) is the interaction of the transcription factor SRF with the co-factor protein Mal in T cells that can be triggered by overexpression of the viral oncoprotein Tip through Tip-induced Rho GTPase activation. Another viral regulator of host transcriptional processes is Tax-1, encoded by human T cell lymphotrophic virus type-1 (HTLV-1). Andrea Kress (Erlangen, Germany) showed that it induces the expression of the tumor marker fascin and that this is a common mechanism among viral oncogenes of lymphotrophic viruses [48]. Bacterial factors, however, are also able to modulate the signalling pathways of their host cells and thus modify the resulting immune response. Dagmar Hildebrand (Heidelberg, Germany) discussed how the Pasteurella multocida protein toxin manipulates cells to produce the pro-inflammatory cytokine IL-1β independently from inflammasome-mediated activation of Caspase-1, a well-characterized pathway described for other pathogens [49]. Bernd Schmeck (Marburg, Germany) highlighted in his talk the fact that the expression of small, non-coding RNAs (miRNA) that are able to repress specific target genes [50] plays an important role for the expression of Legionella pneumophila-induced induction of pro-inflammatory genes and that TLR-receptor-induced signalling cascades are important components in the regulation of miRNA expression [51].

The workshop “Growth Factors, Cytokines and Chemokines” started with a review by Marcus Thelen’s (Bellinzona, Switzerland) on signalling events triggered by atypical chemokine receptors that do not bind to heterotrimeric G proteins [52]. A common theme among the following talks was the control of cell cycle progression. Katja Handschick (Giessen, Germany) presented data on the effects of a constitutively active cyclin-dependent kinase 6 (CDK6) mutant in non-synchronised HeLa cells. With this and other approaches, she could show that gene expression induced by IL-1/TNFα utilises classical cell cycle-regulated pathways. Malte Kriegs (Hamburg, Germany) presented data on the different molecular mechanism of cell cycle arrest in tumor cells induced by radiation or pharmacological EGF receptor inhibitors. These inhibitors have the capacity to enhance the radiation-induced, persistent G1 arrest cells. Thus, inhibition of EGFR, a known key-player in cancer [53] is a promising target for therapeutical sensitisation of tumours displaying an intact p53 pathway after X-ray treatment [54]. Sandip Kar (Heidelberg, Germany) explained that not only qualitative but also quantitative aspects of signalling pathways have an influence on the outcome of cellular signalling. He was able to show that the differential activation of the PI3K/Akt pathway in response to stimulation with erythropoietin can be explained by the different expression levels of PI3K/Akt pathway components, such as the phosphatases SHIP-1 and PTEN.

The last workshop of the conference addressed the topic “Cell Differentiation, Senescence and Death”. Keynote speakers of this session were Timm Schroeder (Munich, Germany) and Peter ten Dijke (Leiden, Netherlands). In his talk, Timm Schroeder impressively demonstrated the importance of single cell, real-time analysis for the investigation of signal transduction pathway activity or the differentiation activity of stem cells [55]. Peter ten Dijke summarised the role of TGFβ signal transduction pathways in cancer [56]. FRET experiments done by Simon Neumann (Stuttgart, Germany) showed that TNF-related apoptosis-inducing ligand (TRAIL) receptors can form heteromeric receptor complexes in the absence of ligand. The group of Ingo Schmitz (Braunschweig, Germany) investigated the expression of the transcription factor Foxp3 which is essential for the development of regulatory T cells (Tregs). They could show that the atypical NFκB inhibitor IκB_NS_ plays an important role in Foxp3 gene transcription and in the absence of IκB_NS_ only a reduced number of Tregs is generated. For his presentation, Ingo Schmitz was awarded the STS Science Award 2012 sponsored by Biomol GmbH, Hamburg. Björn Stork (Düsseldorf, Germany) finished the session with a talk on the factors Atg13 and FIP200 that can support the induction of cellular autophagy also independently of Ulk1 and Ulk2 kinases under basal and starvation conditions [57].

One special highlight of the STS meetings that deserves mentioning is the traditional “One Minute – One Transparency” session where virtually all posters were presented to the plenum to trigger the following poster viewing and discussion. Once again in 2011, this fun event certainly lowered the thresholds to establishing new networks – this time not short-lived signalling networks but rather longer term scientific networks.

## Figures and Tables

**Figure 5:**
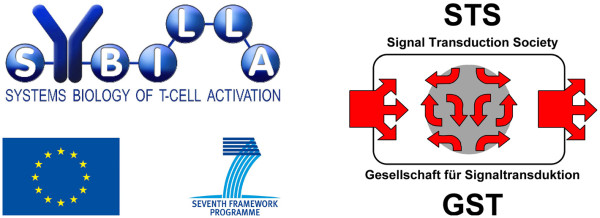
The conference was mainly supported and organized by the EU FP7 consortium SYBILLA (Systemsbiology of T-cell activation) and STS (The Signal Transduction Society).

## References

[B1] Malissen B An evolutionary and structural perspective on T cell antigen receptor function Immunol Rev 2003 191 7 27 10.1034/j.1600-065X.2003.00016.x 12614348

[B2] Alarcon B Gil D Delgado P Schamel WWA Initiation of TCR signaling: regulation within CD3 dimers Immunol Rev 2003 191 38 46 10.1034/j.1600-065X.2003.00017.x 12614350

[B3] Deswal S Schulze AK Hofer T Schamel WW Quantitative analysis of protein phosphorylations and interactions by multi-colour IP-FCM as an input for kinetic modelling of signalling networks PLoS One 2011 6 e22928 10.1371/journal.pone.0022928 21829558 PMC3146539

[B4] Wepf A Glatter T Schmidt A Aebersold R Gstaiger M Quantitative interaction proteomics using mass spectrometry Nat Methods 2009 6 203 205 10.1038/nmeth.1302 19198594

[B5] Dopfer EP Schopf B Louis-Dit-Sully C Dengler E Hohne K Klescova A Prouza M Suchanek M Reth M Schamel WW Analysis of novel phospho-ITAM specific antibodies in a S2 reconstitution system for TCR-CD3 signalling Immunol Lett 2010 130 43 50 10.1016/j.imlet.2009.12.011 20005895

[B6] Feller SM Hass R Janssen O Friedrich K Cell Communication and Signaling is becoming the official journal of the Signal Transduction Society Cell Commun Signal 2008 6 1 10.1186/1478-811X-6-1 18680612 PMC2542370

[B7] Swamy M Siegers GM Fiala GJ Molnar E Dopfer EP Fisch P Schraven B Schamel WW Stoichiometry and intracellular fate of TRIM-containing TCR complexes Cell Commun Signal 2010 8 5 10.1186/1478-811X-8-5 20298603 PMC2848047

[B8] 12th Joint Meeting of the Signal Transduction Society (STS). Signal Transduction: Receptors, Mediators and Genes Weimar, Germany. 29–31 October 2008. Abstracts Cell Commun Signal 2009 7 Suppl 1 A1-A108 19281624 10.1186/1478-811X-7-S1-A1 PMC4291581

[B9] Entschladen F Altschmied J Baumgrass R Behrmann I Giehl K Hermanns H Huber O Kieser A Klotz LO Kubatzky KF Signal transduction, receptors, mediators and genes: younger than ever - the 13th meeting of the Signal Transduction Society focused on aging and immunology Cell Commun Signal 2010 8 2 10.1186/1478-811X-8-2 20181226 PMC2833152

[B10] Davis MM Boniface JJ Reich Z Lyons D Hampl J Arden B Chien Y Ligand recognition by alpha/beta T cell receptors Annu Rev Immunol 1998 16 523 544 10.1146/annurev.immunol.16.1.523 9597140

[B11] Lettau M Pieper J Janssen O Nck adapter proteins: functional versatility in T cells Cell Commun Signal 2009 7 1 10.1186/1478-811X-7-1 19187548 PMC2661883

[B12] Gil D Schamel WW Montoya M Sanchez-Madrid F Alarcon B Recruitment of Nck by CD3 epsilon reveals a ligand-induced conformational change essential for T cell receptor signaling and synapse formation Cell 2002 109 901 912 10.1016/S0092-8674(02)00799-7 12110186

[B13] Martinez-Martin N Risueno RM Morreale A Zaldivar I Fernandez-Arenas E Herranz F Ortiz AR Alarcon B Cooperativity between T cell receptor complexes revealed by conformational mutants of CD3epsilon Sci Signal 2009 2 ra43 10.1126/scisignal.2000402 19671929

[B14] Swamy M Dopfer EP Molnar E Alarcon B Schamel WW The 450 kDa TCR Complex has a Stoichiometry of abgedezz Scand J Immunol 2008 67 418 420 author reply 421 10.1111/j.1365-3083.2008.02082.x 18282230

[B15] Arechaga I Swamy M Abia D Schamel WA Alarcon B Valpuesta JM Structural characterization of the TCR complex by electron microscopy Int Immunol 2010 22 897 903 10.1093/intimm/dxq443 21059766

[B16] Schamel WW Arechaga I Risueno RM van Santen HM Cabezas P Risco C Valpuesta JM Alarcon B Coexistence of multivalent and monovalent TCRs explains high sensitivity and wide range of response J Exp Med 2005 202 493 503 10.1084/jem.20042155 16087711 PMC2212847

[B17] Lillemeier BF Mortelmaier MA Forstner MB Huppa JB Groves JT Davis MM TCR and Lat are expressed on separate protein islands on T cell membranes and concatenate during activation Nat Immunol 2010 11 90 96 20010844 10.1038/ni.1832 PMC3273422

[B18] Kumar R Ferez M Swamy M Arechaga I Rejas MT Valpuesta JM Schamel WW Alarcon B van Santen HM Increased sensitivity of antigen-experienced T cells through the enrichment of oligomeric T cell receptor complexes Immunity 2011 35 375 387 10.1016/j.immuni.2011.08.010 21903423

[B19] Simons K Toomre D Lipid rafts and signal transduction Nat Rev Mol Cell Biol 2000 1 31 39 10.1038/35036052 11413487

[B20] Harder T Sangani D Plasma membrane rafts engaged in T cell signalling: new developments in an old concept Cell Commun Signal 2009 7 21 10.1186/1478-811X-7-21 19732448 PMC2744677

[B21] Kennedy C Nelson MD Bamezai AK Analysis of Detergent-free Lipid Rafts isolated from a CD4+ T cell line: Interaction with antigen presenting cells promotes coalescing of lipid rafts Cell Commun Signal 2011 9 31 10.1186/1478-811X-9-31 22151974 PMC3283486

[B22] Feller SM First Honorary Medal of the Signal Transduction Society (STS) and 'CELL COMMUNICATION AND SIGNALING' awarded to Professor Anthony J. (Tony) Pawson Cell Commun Signal 2011 9 3 10.1186/1478-811X-9-3 21247454 PMC3032762

[B23] Eckhart W Hutchinson MA Hunter T An activity phosphorylating tyrosine in polyoma T antigen immunoprecipitates Cell 1979 18 925 933 10.1016/0092-8674(79)90205-8 229973

[B24] Nika K Soldani C Salek M Paster W Gray A Etzensperger R Fugger L Polzella P Cerundolo V Dushek O Constitutively active Lck kinase in T cells drives antigen receptor signal transduction Immunity 2010 32 766 777 10.1016/j.immuni.2010.05.011 20541955 PMC2996607

[B25] Kobayashi H Azuma R Yasunaga T Expression of excess receptors and negative feedback control of signal pathways are required for rapid activation and prompt cessation of signal transduction Cell Commun Signal 2009 7 3 10.1186/1478-811X-7-3 19254388 PMC2666736

[B26] Altan-Bonnet G Germain RN Modeling T cell antigen discrimination based on feedback control of digital ERK responses PLoS Biol 2005 3 e356 10.1371/journal.pbio.0030356 16231973 PMC1262625

[B27] Dushek O Aleksic M Wheeler RJ Zhang H Cordoba SP Peng YC Chen JL Cerundolo V Dong T Coombs D van der Merwe PA Antigen potency and maximal efficacy reveal a mechanism of efficient T cell activation Sci Signal 2011 4 ra39 10.1126/scisignal.2001430 21653229 PMC4143974

[B28] Risueno RM Schamel WW Alarcon B T cell receptor engagement triggers its CD3epsilon and CD3zeta subunits to adopt a compact, locked conformation PLoS One 2008 3 e1747 10.1371/journal.pone.0001747 18320063 PMC2254190

[B29] Minguet S Swamy M Alarcon B Luescher IF Schamel WW Full activation of the T cell receptor requires both clustering and conformational changes at CD3 Immunity 2007 26 43 54 10.1016/j.immuni.2006.10.019 17188005

[B30] Palmer E Naeher D Affinity threshold for thymic selection through a T-cell receptor-co-receptor zipper Nat Rev Immunol 2009 9 207 213 10.1038/nri2469 19151748

[B31] McKeithan TW Kinetic proofreading in T-cell receptor signal transduction Proc Natl Acad Sci U S A 1995 92 5042 5046 10.1073/pnas.92.11.5042 7761445 PMC41844

[B32] Mingueneau M Roncagalli R Gregoire C Kissenpfennig A Miazek A Archambaud C Wang Y Perrin P Bertosio E Sansoni A Loss of the LAT adaptor converts antigen-responsive T cells into pathogenic effectors that function independently of the T cell receptor Immunity 2009 31 197 208 10.1016/j.immuni.2009.05.013 19682930

[B33] Gruber T Hermann-Kleiter N Hinterleitner R Fresser F Schneider R Gastl G Penninger JM Baier G PKC-theta modulates the strength of T cell responses by targeting Cbl-b for ubiquitination and degradation Sci Signal 2009 2 ra30 10.1126/scisignal.2000046 19549985

[B34] Hermann-Kleiter N Gruber T Lutz-Nicoladoni C Thuille N Fresser F Labi V Schiefermeier N Warnecke M Huber L Villunger A The nuclear orphan receptor NR2F6 suppresses lymphocyte activation and T helper 17-dependent autoimmunity Immunity 2008 29 205 216 10.1016/j.immuni.2008.06.008 18701084 PMC4941926

[B35] Elo LL Jarvenpaa H Tuomela S Raghav S Ahlfors H Laurila K Gupta B Lund RJ Tahvanainen J Hawkins RD Genome-wide profiling of interleukin-4 and STAT6 transcription factor regulation of human Th2 cell programming Immunity 2010 32 852 862 10.1016/j.immuni.2010.06.011 20620947

[B36] Contento RL Campello S Trovato AE Magrini E Anselmi F Viola A Adhesion shapes T cells for prompt and sustained T-cell receptor signalling Embo J 2010 29 4035 4047 10.1038/emboj.2010.258 20953162 PMC3020646

[B37] Saez-Rodriguez J Simeoni L Lindquist JA Hemenway R Bommhardt U Arndt B Haus UU Weismantel R Gilles ED Klamt S Schraven B A logical model provides insights into T cell receptor signaling PLoS Comput Biol 2007 3 e163 10.1371/journal.pcbi.0030163 17722974 PMC1950951

[B38] Beyer T Busse M Hristov K Gurbiel S Smida M Haus UU Ballerstein K Pfeuffer F Weismantel R Schraven B Lindquist JA Integrating signals from the T-cell receptor and the interleukin-2 receptor PLoS Comput Biol 2011 7 e1002121 10.1371/journal.pcbi.1002121 21829342 PMC3150289

[B39] Schmidt H Gelhaus C Nebendahl M Lettau M Watzl C Kabelitz D Leippe M Janssen O 2-D DIGE analyses of enriched secretory lysosomes reveal heterogeneous profiles of functionally relevant proteins in leukemic and activated human NK cells Proteomics 2008 8 2911 2925 10.1002/pmic.200800170 18655029

[B40] Schmidt H Gelhaus C Nebendahl M Lettau M Lucius R Leippe M Kabelitz D Janssen O Effector granules in human T lymphocytes: the luminal proteome of secretory lysosomes from human T cells Cell Commun Signal 2011 9 4 10.1186/1478-811X-9-4 21255389 PMC3034720

[B41] Schmidt H Gelhaus C Lucius R Nebendahl M Leippe M Janssen O Enrichment and analysis of secretory lysosomes from lymphocyte populations BMC Immunol 2009 10 41 10.1186/1471-2172-10-41 19640298 PMC2726124

[B42] Schmidt H Gelhaus C Nebendahl M Lettau M Lucius R Leippe M Kabelitz D Janssen O Effector granules in human T lymphocytes: proteomic evidence for two distinct species of cytotoxic effector vesicles J Proteome Res 2011 10 1603 1620 10.1021/pr100967v 21247065

[B43] Haan C Rolvering C Raulf F Kapp M Druckes P Thoma G Behrmann I Zerwes HG Jak1 has a dominant role over Jak3 in signal transduction through gammac-containing cytokine receptors Chem Biol 2011 18 314 323 10.1016/j.chembiol.2011.01.012 21439476

[B44] Demaria M Poli V From the nucleus to the mitochondria and back: the odyssey of a multitask STAT3 Cell Cycle 2011 10 3221 3222 10.4161/cc.10.19.17379 21926478

[B45] Wu Z Doondeea JB Gholami AM Janning MC Lemeer S Kramer K Eccles SA Gollin SM Grenman R Walch A Quantitative chemical proteomics reveals new potential drug targets in head and neck cancer. Mol Cell Proteomics 2011 10 M111 011635 10.1074/mcp.M111.011635 PMC3237086 21955398

[B46] Pan X Rudolph JM Abraham L Habermann A Haller C Krijnse-Locker J Fackler OT HIV-1 Nef compensates for disorganization of the immunological synapse by inducing trans-Golgi network-associated Lck signaling Blood 2012 119 786 797 10.1182/blood-2011-08-373209 22123847

[B47] McLaughlin-Drubin ME Munger K Viruses associated with human cancer Biochim Biophys Acta 2008 1782 127 150 10.1016/j.bbadis.2007.12.005 18201576 PMC2267909

[B48] Kress AK Kalmer M Rowan AG Grassmann R Fleckenstein B The tumor marker Fascin is strongly induced by the Tax oncoprotein of HTLV-1 through NF-kappaB signals Blood 2011 117 3609 3612 10.1182/blood-2010-09-305805 21300980

[B49] Muller A Oertli M Arnold IC H. pylori exploits and manipulates innate and adaptive immune cell signaling pathways to establish persistent infection Cell Commun Signal 2011 9 25 10.1186/1478-811X-9-25 22044597 PMC3214186

[B50] Davis BN Hata A Regulation of MicroRNA Biogenesis: A miRiad of mechanisms Cell Commun Signal 2009 7 18 10.1186/1478-811X-7-18 19664273 PMC3224893

[B51] O'Neill LA Sheedy FJ McCoy CE MicroRNAs: the fine-tuners of Toll-like receptor signalling Nat Rev Immunol 2011 11 163 175 10.1038/nri2957 21331081

[B52] Thelen M Stein JV How chemokines invite leukocytes to dance Nat Immunol 2008 9 953 959 10.1038/ni.f.207 18711432

[B53] Seufferlein T Ahn J Krndija D Lother U Adler G von Wichert G Tumor biology and cancer therapy - an evolving relationship Cell Commun Signal 2009 7 19 10.1186/1478-811X-7-19 19678929 PMC2731766

[B54] Wang M Morsbach F Sander D Gheorghiu L Nanda A Benes C Kriegs M Krause M Dikomey E Baumann M EGF receptor inhibition radiosensitizes NSCLC cells by inducing senescence in cells sustaining DNA double-strand breaks Cancer Res 2011 71 6261 6269 10.1158/0008-5472.CAN-11-0213 21852385 PMC3185115

[B55] Schroeder T Long-term single-cell imaging of mammalian stem cells Nat Methods 2011 8 S30 35 10.1038/nmeth.1577 21451514

[B56] Meulmeester E Ten Dijke P The dynamic roles of TGF-beta in cancer J Pathol 2011 223 205 218 20957627 10.1002/path.2785

[B57] Alers S Löffler AS Paasch F Dieterle AM Keppeler H Lauber K Campbell DG Fehrenbacher B Schaller M Wesselborg S Stork B Atg13 and FIP200 act independently of Ulk1 and Ulk2 in autophagy induction. Autophagy 2011 7 Epub ahead of print 10.4161/auto.7.12.18027 PMC3327613 22024743

